# Effectiveness of smoking-cessation interventions for urban hospital patients: study protocol for a randomized controlled trial

**DOI:** 10.1186/1745-6215-13-126

**Published:** 2012-08-01

**Authors:** Ellie Grossman, Donna Shelley, R Scott Braithwaite, Iryna Lobach, Ana Goffin, Erin Rogers, Scott Sherman

**Affiliations:** 1 Division of General Internal Medicine, New York University School of Medicine, New York, NY, USA; 2 Bellevue Hospital Center, New York, NY, USA; 3 New York University College of Dentistry, New York, NY, USA; 4 Department of Environmental Medicine, New York University School of Medicine, New York, NY, USA; 5 VA New York Harbor Healthcare System, New York, NY, USA

**Keywords:** Hospital, Randomized trial, Smoking cessation, Telephone counseling, Underserved population

## Abstract

**Background:**

Hospitalization may be a particularly important time to promote smoking cessation, especially in the immediate post-discharge period. However, there are few studies to date that shed light on the most effective or cost-effective methods to provide post-discharge cessation treatment, especially among low-income populations and those with a heavy burden of mental illness and substance use disorders.

**Methods/design:**

This randomized trial will compare the effectiveness and cost-effectiveness of two approaches to smoking cessation treatment among patients discharged from two urban public hospitals in New York City. During hospitalization, staff will be prompted to ask about smoking and to offer nicotine replacement therapy (NRT) on admission and at discharge. Subjects will be randomized on discharge to one of two arms: one arm will be proactive multi-session telephone counseling with motivational enhancement delivered by study staff, and the other will be a faxed or online referral to the New York State Quitline. The primary outcome is 30-day point-prevalence abstinence from smoking at 6-month follow-up post-discharge. We will also examine cost-effectiveness from a societal and a payer perspective, as well as explore subgroup analyses related to patient location of hospitalization, race/ethnicity, immigrant status, and inpatient diagnosis.

**Discussion:**

This study will explore issues of implementation feasibility in a post-hospitalization patient population, as well as add information about the effectiveness and cost-effectiveness of different strategies for designing smoking cessation programs for hospitalized patients.

**Trial registration:**

Clinicaltrials.gov ID# NCT01363245

## Background

Despite 40 years of progress in reducing the prevalence of smoking, it remains the leading preventable cause of death in the United States. Hospitals are an important place to intervene, particularly for smokers of lower socioeconomic status. Studies have shown follow-up after hospital discharge to be efficacious in helping smokers to quit, but there are still many questions about how to structure the follow-up and how effective it is in actual practice.

While smoking prevalence in the US has declined from 24.1% in 1998 to 20.6% in 2008, it has not declined consistently across all segments of the population [1]. There are large discrepancies by level of income and education, as well as by racial and ethnic-minority status [2]. High-risk groups are less likely to receive advice from a provider to quit smoking, to use proven treatment, or to quit successfully, compared with white smokers and smokers above the poverty line [3].

Public safety net hospital systems are the main source of care for many patients of lower socioeconomic status. Developing programs to improve smoking cessation outcomes in these underserved populations is an essential step toward achieving national health objectives and should help reduce tobacco-related health disparities by targeting the most economically disadvantaged.

Hospitalized patients, particularly in psychiatric units, have a higher prevalence of smoking than the general population. It is a time of enforced abstinence and heightened vulnerability, making it a unique opportunity to intervene. Of the few studies examining smoking cessation among hospitalized patients, most have been efficacy studies, using dedicated, specially trained smoking cessation staff, with significant additional resources and applying narrow eligibility criteria [4]. No studies have included an entire hospital in a system-wide intervention with follow-up post-discharge.

While post-discharge counseling needs to be provided for at least 4 weeks after discharge [4], there is little evidence about best practices in offering such post-discharge services. Telephone counseling via Quitlines is available in all 50 states, has broad reach, and is very effective [3,5]. However, statewide Quitlines are seldom used within health care [6], both because facilities seldom refer to telephone counseling [7] and patients referred seldom follow through [8]. Quitlines in 49 US states allow health care providers to refer patients online or via fax for proactive telephone counseling [9], which may be a feasible and low-cost way to enroll smokers in counseling [10-12]. Few studies have examined Quitlines as a resource for hospitalized or recently hospitalized patients. In a study of surgical patients, referral to a Quitline for post-discharge proactive cessation counseling was found to be feasible, acceptable to surgical patients and staff, and inexpensive [13].

Although referral to a Quitline may be an inexpensive method of ensuring post-discharge follow-up for hospitalized smokers, its use in this setting has never been compared to a more intensive telephone counseling system. In this trial, we aim to compare a more intensive telephone counseling system to a simple referral to the state Quitline. The more intensive counseling system, while likely to be more expensive for a hospital system to implement than referral to the Quitline, may be more cost-effective if it results in a higher quit rate. Post-discharge patients may particularly benefit from a more intensive counseling approach, and they may require more or different attempts to make contact and maintain treatment engagement than is feasible for the Quitline.

## Methods/design

### Overview

We plan to perform a randomized controlled trial comparing the effectiveness and cost-effectiveness of two approaches to smoking-cessation counseling after discharge from the hospital. We will enroll smokers from all inpatient units at two urban hospitals serving predominantly low-income populations (Bellevue Hospital Center and the Veterans Affairs (VA) New York Harbor Healthcare System). During hospitalization, all patients will receive usual care: 1) screening, 2) brief counseling by the nurse and/or doctor, and 3) access to nicotine-replacement therapy (NRT). All patients will be offered 4 weeks of NRT on discharge and will be randomized to one of two arms for post-discharge counseling: 1) intensive, proactive telephone counseling (seven sessions over 6 to 8 weeks) from a service affiliated with the patient’s hospital, or 2) faxed or online referral to the New York State Quitline for one proactive counseling session, followed by reactive counseling if patients call the Quitline for additional sessions. The primary outcome is smoking abstinence at 6-month follow-up post-discharge.

### Setting

The two hospitals where we are performing our study are the following:

1. Bellevue Hospital Center, the nation’s oldest public hospital, is a core New York University School of Medicine teaching facility serving a very diverse medically underserved population. There are approximately 20,000 admissions/year to adult medical/surgical services, and 4,000 admissions to adult psychiatric units. Limited English proficiency is common (50 to 60%), and 40% of inpatients are Hispanic, 30% African American, 10% Asian and 17% Caucasian. The prevalence of smoking is 25% for inpatient non-psychiatric services and 55 to 60% for the psychiatric units. Among the smokers, the language breakdown is: English 48%, Spanish 27%, Mandarin 13%, Cantonese 8%, and other/unknown 4%. One in three patients is uninsured, and this number is increasing (by 8% from 2007 to 2008). Among inpatients, 60% have Medicaid, 15% Medicare, and 8% commercial insurance.

2. The Manhattan campus of the VA New York Harbor Healthcare System (Manhattan VA), also a core New York University teaching facility, serves primarily low income veterans in Manhattan and the surrounding areas. There are approximately 4,000 admissions/year to the medical/surgical services and 1,000 to psychiatry. Approximately 60% of inpatients are Caucasian, 31% are African American, and 17% are Hispanic or Latino; most (61%) have a high school education or less.

Both sites use a fully electronic medical record (EMR) system [14,15]. The EMRs at both sites include a wide range of clinical reminders to increase adherence to practice guidelines (including smoking cessation), and the level of guideline adherence is measured routinely [16-18].

### Participants

**Figure 1:**
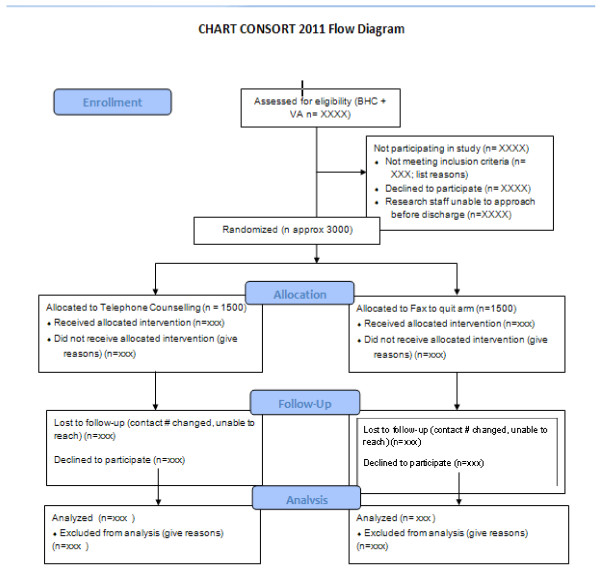
** Consortium of Hospitals Advancing Research on Tobacco** (**CHART) CONSORT 2011 flow diagram.**

We plan to recruit all adult smokers admitted to the two hospitals (see Figure 1 for flow diagram). Inclusion criteria are: 1) age ≥ 18 years, 2) smoked tobacco during the prior 30 days, 3) have an active US phone number, and 4) able to provide consent in English, Spanish or Mandarin (Table 1). We chose these three languages as it will allow us to approach nearly 90% of smokers at Bellevue and all smokers at the Manhattan VA. Patients will be excluded if they use only smokeless tobacco or products such as betel, are pregnant or breastfeeding, are discharged to an institution (for example, jail/prison, nursing home, long-term psychiatric facility), or do not have the cognitive or physical ability to enroll or participate in the study.

**Table 1:** Eligibility criteria

**Inclusion**	**Exclusion criteria - patients will be excluded if they:**
Age ≥ 18 years*	Use only smokeless tobacco or products such as betel
Smoked tobacco during the prior 30 days*	Are pregnant or breastfeeding
Have an active US phone number	Are discharged to an institution (for example, jail/prison, nursing home, long-term psychiatric facility)
Able to provide consent in English, Spanish, or Mandarin	Do not have the cognitive or physical ability to enroll or participate in the study*

*Common to all Consortium of Hospitals Advancing Research on Tobacco (CHART) trials.

### Outcome measures

Our primary outcome will be 30-day point-prevalence abstinence from smoking, as measured at 6-month follow-up post-discharge via participant self-report. We will perform this assessment via telephone survey. Our hypotheses are that, compared to control (Quitline), multisession phone counseling patients will have higher rates of smoking cessation, lower cigarette consumption, and more quit attempts.

Secondary outcomes are biochemically validated abstinence from smoking at 6-month follow-up (as measured by salivary cotinine mailed in for collection via a protocol described more fully elsewhere [19]) and smoking abstinence at 12-month post-discharge follow-up (as measured by participant self-report on telephone survey). For pre-specified subgroup analyses, we will compare outcomes by participant race/ethnicity, immigrant status, inpatient diagnosis, and location of hospitalization.

As an exploratory sub-study, we will examine patient use of text messaging and interest in a cessation intervention using this technology. Given the mobility and relative youth of our population, text messaging could be an effective way to reach these patients. The aims of this exploratory study are to assess: 1) the prevalence of regular text messaging use, and 2) interest in counseling delivered via text messaging.

### Cost-effectiveness outcomes

As a companion to our effectiveness study, we will compare the cost-effectiveness of the two intervention arms from both a societal and a payer perspective. Our hypothesis is that the intensive telephone counseling intervention will have incremental cost-effectiveness ratios consistent with current standards of health care value in the US.

### Co-variables of interest

Covariables of interest are found in Table 2. Among these variables will be sociodemographic measures; measures of nicotine addiction, smoking cessation history, and interest in quitting; comorbid mood symptoms and substance use history, and medical history. We will also collect information about the index hospitalization, including length of stay, principal diagnoses, and procedure codes.

**Table 2:** Data collection items and schedule

**Measure**	**Baseline**	**2-Month follow-up**	**4-Month follow-up**	**6-Month follow-up**	**9-Month follow-up**	**12-Month follow-up**
***Survey measures***						
*Socio-demographics	X					
*Smoking: history, current use, readiness to quit, quit attempts, use of medications or other treatments	X	X	X	X	X	X
Health habits: alcohol, substance abuse	X			X		X
Health status (HUI 3, EQ5D), medical conditions, health-care utilization	X			X		X
Depression (PHQ-2)	X			X		X
Text messaging preferences	X					
***Other measures***						
*Biochemical validation				X		
Satisfaction with intervention components		X				
Intervention fidelity, including contact with Quitline		X		X		X

*These measures (at least in part) are common to all Consortium of Hospitals Advancing Research on Tobacco (CHART) Trials.

### Facilitating participant retention

**Figure 2:**
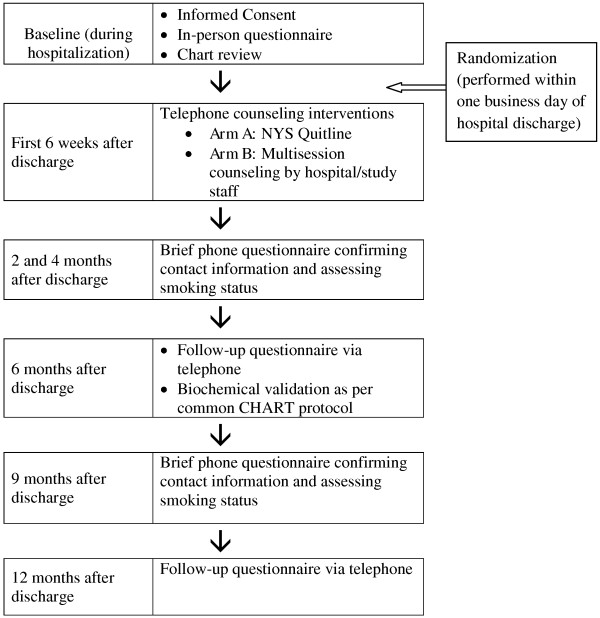
Schedule for intervention contacts and follow-up assessments.

Performing a post-discharge intervention and assessing follow-up smoking status may be particularly difficult in this highly mobile, difficult-to-reach patient population. On enrollment into the study, participants will be asked to provide their own contact information (including multiple phone numbers) and also contact information for two friends or family members who are likely to remain in contact with them if they change their residence. To facilitate retention in the counseling intervention, counselors will arrange the scheduled phone calls at times and phone numbers that are convenient for the patients. We will perform brief interim follow-up assessments via phone at 2, 4, and 9 months post-discharge to confirm contact information and assess smoking status (see Figure 2 for overall study timeline). If we cannot reach a participant after three attempts at any given time point for a follow-up assessment, we will call the alternate contact people given at enrollment. If needed, we will also use participant email contact information as a means to arrange phone communication. For participants we are still unable to contact, we will check the hospital system’s medical record to try to find any additional information.

### Interventions

#### A. During hospitalization – usual care

Usual care includes screening, educational materials, and access to NRT. We will provide at least two 1-hour training sessions for nurses and physicians (residents and staff), covering both content (evidence for treatment) and process (how and when to treat) – with periodic brief refresher sessions. More importantly, the EMR will provide guidance about treatment and reminders to assess smoking status and treat as appropriate (for example, with a link to ‘order entry’ to prescribe NRT when a physician indicates that the patient has recently smoked), since we have found that to be a more useful approach to training [20]. This will help ensure all smokers receive brief advice to quit while hospitalized.

On enrollment to the study, patients will receive information about local cessation resources, NRT, and the study itself, so they can read it while hospitalized. The NRT information will include a troubleshooting number to call in case of problems obtaining medication. We do not expect many patients to have difficulty obtaining NRT, but we want to ensure all patients have access to it. Study staff will also provide information about local cessation resources to all screened patients who do not enroll in the study.

#### B. On discharge

##### B1. Discharge medications

An EMR notification and/or physician-directed flyer at the patient bedside will remind physicians to provide a prescription for at least 4 weeks of NRT or other smoking cessation medication on discharge (for all patients, whether enrolled or not). Patients may receive any form of medication at the discretion of the prescribing physician. Bellevue patients without insurance will fill their discharge prescriptions at the Bellevue discharge pharmacy (which has NRT patch and gum and bupropion SR on formulary). Bellevue patients with Medicaid or other insurance will fill their prescriptions at pharmacies in their local areas; they may have co-pays, which will vary according to insurance type. VA patients will obtain their medication via the VA pharmacy (which has NRT patch/gum/lozenge, bupropion SR and varenicline on formulary).

For patients enrolled in the study, counselors in both arms of the study will assist patients in obtaining 4 weeks of NRT patch or gum after discharge if they have encountered problems and/or need help. In the ‘telephone counseling’ arm, we have an algorithm that will help counselors suggest appropriate NRT regimens depending on patient tobacco use patterns. Again, the manner of obtaining the NRT will vary, depending on hospital and insurance status. For Bellevue patients with Medicaid, a study physician will write an appropriate prescription and mail it to the patient’s home for them to fill at an outside pharmacy. For Bellevue patients with private insurance, Medicare, or no insurance, study staff will generate an order at drugstore.com, which will send medication to the patient free of charge. For VA patients, a study physician will write an appropriate prescription and the VA mail-order pharmacy will send the medication to the patient’s home as per VA outpatient pharmacy protocol. Patients in the Quitline arm will have access to NRT as per usual Quitline protocol.

##### B2. Discharge information

For all patients (whether enrolled or not), computerized patient discharge instructions will include encouragement to quit smoking and information about local resources. Participating patients will have received additional information provided on enrollment (as described above).

#### C. Post-discharge

##### C1. Quitline arm

In January 2000, New York State launched a Smoker’s Quitline (located at Roswell Park Cancer Institute, Buffalo, NY, USA) providing counseling in over 100 languages, with quit rates comparable [21] to those found in published reports. The Quitline provides a proactive counseling service referred to as the “Refer-to-Quit” program. For patients in the Quitline arm, the Research Assistant will fax a referral or submit it electronically at the time of randomization (shortly after discharge). The Quitline will make up to five calls at different times of the day to reach the patient. Once contacted, the patient receives one 15 to 20 minute telephone counseling session and a follow-up call to assess quit status (and check that medication was received if it had been requested). Patients can call the Quitline for additional follow-up sessions.

##### C2. Telephone counseling arm

Patients in the Telephone Counseling arm will receive telephone calls from study staff at days and times convenient for the patient. To establish the first post-discharge contact, staff will call each patient up to 10 times during the 2 weeks following discharge. Once contact has been made, the staff will complete tailored, multi-session telephone counseling with the patients.

Our telephone counseling system includes proactive counseling, a structured protocol, and relapse-sensitive scheduling [22]. The content is based on Motivational Interviewing and Problem Solving Therapy and addresses behavioral and cognitive issues, including motivation, self-efficacy, difficult situations, comorbid symptoms, coping strategies, medication usage, and relapse prevention. The program staff members use a structured protocol to maintain a record of each of the counseling calls for internal quality assurance.

### The planning session

The first counseling session lasts approximately 20 to 30 minutes and helps patients develop an individualized plan to quit smoking or to remain abstinent (for those patients who have already quit). Content areas include smoking and quitting history, motivation, environmental factors, planning, proper use of quitting aids, and setting a quit date if needed.

### Follow-up sessions

Follow-up calls will be weekly until a quit date is set and at 1, 3, 7, 14, 30, and 42 days after each patient’s quit date. The calls are intended to prevent relapse and to help those who relapse resume quitting. Follow-up calls (10 to 15 minutes) also use a counselors’ manual for consistency and fidelity monitoring. Subjects are able to call the program staff in between scheduled calls if desired.

### Randomization

We will randomize participants to one of the two arms of the trial in a 1:1 ratio shortly after discharge, when information regarding discharge location becomes available in the medical record. Participants who have enrolled in the study while they are in the hospital, but who are then discharged to a nursing home or other facility where smoking is forbidden and patients cannot leave to smoke, will be deemed ineligible and will not undergo randomization. Allocation concealment will be ensured, as the randomization code will not be released until the patients are recruited into the trial and all baseline measures are taken. We arranged our complete randomization scheme before the study began, given the expected number of participants from our power and sample size analysis. The randomization was performed using routines for random number generation available in R Package for Scientific Computing (http://www.r-project.org). To prevent a small number of patients in a particular study site from all receiving, by chance, the same treatment, treatment assignment was randomized with stratification on the study site (Bellevue versus Manhattan VA). Study staff who perform follow-up assessments are blinded to participants’ intervention assignment, as are those performing the analyses of saliva cotinine. The trial statistician performing the analyses of all study data is masked to the treatment allocation.

### Intervention standardization and fidelity

The Quitline has a standardized protocol and has well-trained counselors answering its telephone calls and providing counseling. For the Telephone Counseling arm, the telephone counselors will undergo an intensive initial training consisting of a) 5 hours of Motivational Interviewing training with a clinical psychologist specializing in health psychology, and b) 20 to 30 hours of training on the study’s clinical protocols. This training will include role-plays with each other and with the study’s counselor supervisor. After the counselor supervisor has determined that a counselor has met the role-play training objectives, the counselor will advance to complete a series of standardized patient (SP) encounters. SP encounters will involve calling an actor trained to portray a smoker enrolled in the study and completing planning and follow-up counseling sessions per study protocol. The SP encounters will be audiotaped and reviewed by a clinical psychologist and the study’s counselor supervisor for adherence to trial protocols. Each counselor will complete two to four SP encounters, depending on their training needs as determined by the study’s counselor supervisor.

To ensure intervention standardization and fidelity after study implementation, a random sample of the counselors’ phone calls will be audiotaped and reviewed by a clinical psychologist and the study’s counselor supervisor. Feedback on the calls will be given to counselors verbally and documented using a standardized form with scales indicating whether the counselor adhered to Motivational Interviewing counseling techniques and met the appropriate clinical objectives. Counselors will also attend weekly group supervision meetings with a clinical psychologist, during which time the counselors’ active cases will be discussed with particular attention to difficult cases.

### Sample size

We will compare the 6-month abstinence rates of the two arms of our trial. There are approximately 4,000 ‘current smokers’ admitted to Bellevue non-psychiatric services and 2,400 admitted to psychiatric services per year. We anticipate enrolling 20% of the non-psychiatric patients and 10% of the psychiatric patients – netting a total enrollment of 1,040 Bellevue patients per year of the study. At the VA, there are approximately 1,200 ‘current smokers’ admitted to non-psychiatric services and 500 admitted to psychiatric services per year. With a similar anticipated enrollment rate, our projected enrollment at the VA is 290 patients per year of the study.

In total, we expect to enroll a total of approximately 3,000 patients over the 2.5 years of enrollment, or 1,500 per arm. We expect that the long-term abstinence rate in the Quitline arm will be 5%. With 1,500 participants in each treatment arm, the two-sided unpooled z-test of proportions will have 80% power to detect 2.47% difference in abstinence rates between the two arms at the 0.05 significance level, meaning detecting an increase in cessation rate to 7.47%.

### Data analysis

#### Primary outcome - aim 1

Compare the effectiveness of two post-discharge models of care: 1) Quitline referral, and 2) multi-session telephone counseling.

Descriptive analysis techniques will be used to present baseline characteristics of participants in each treatment arm across the two centers and stratified by center. We will use tests of means and proportions to compare characteristics of the treatment groups and log-linear tests to investigate treatment by center interaction. If the differences between the centers are not significant, the subsequent comparison of the primary outcome will be performed between the two treatment groups across the centers, otherwise it will be stratified on center. Baseline characteristics of participants who drop out will be compared with the other participants to examine whether the drop out is at random. In the case of differential dropout rates (for example, between treatment arms, between centers), we will further examine the factors that led to the drop out. The two-sided z-test of proportions will be used to compare the rates of abstinence from smoking at 6-month follow-up of the two treatment arms.

#### Primary outcome - aim 2

Cost-effectiveness analysis.

Following accepted standards for cost-effectiveness analysis in health care [23], we will perform analyses from a societal perspective (all expenditures, regardless of whether from payers, patients or other sources). We will also perform sensitivity analyses from a payer perspective, as this perspective is sometimes preferred by decision makers. Costs will be discounted at a rate of 3%. Outcomes will be expressed both in terms of cost per quality-adjusted life year (QALY) and cost per life year.

Data inputs will include differential quit rates, along with measures of their uncertainty. To estimate their impact, we will develop a Markov model that will predict differential incidence and impact of cardiovascular and pulmonary disease, based on observed differential quit rates of known duration, effect size, and statistical certitude. In base case analyses, we will conservatively assume that smoking cessation has no beneficial impact on quality of life apart from decreasing the likelihood of these longer-term adverse outcomes, although in sensitivity analyses we can consider various scenarios regarding this effect, including any changes in quality of life. We will also explore different assumptions about duration of intervention effect.

Cost inputs will include the costs of staff time for the in- and out-of-hospital components, including additional staff training required to carry out the intervention. Staff will prospectively track time trying to call each patient and the duration of each counseling call (to value the patient time-costs of participating). We will track pharmacy expenditures for smoking-cessation medications. We will use cigarette smoking frequency to estimate cost-savings to patients from decreased cigarette consumption. Finally, our analyses will include the potential for downstream cost-savings (reduced incidence of cardiovascular and pulmonary disease) and cost increase (additional medical care).

By comparing incremental costs to incremental benefits over a lifetime horizon, we will estimate the value of this intervention, enabling its value to be compared to alternative resource uses. In addition, we will compare incremental cost-effectiveness ratios to accepted standards for favorable value in the US ($100,000 per life-year, and/or $100,000 per QALY).

### Secondary outcomes

We will use a similar approach for the secondary outcomes, to explore the relationship of the intervention’s effectiveness to specific patient characteristics. We will investigate interaction between treatment and patient race/ethnicity, immigrant status, hospital and type of inpatient diagnosis (medical versus surgical versus psychiatric). The interaction analysis will be performed using log-linear tests and analysis of variance (ANOVA) techniques. Assumptions will be checked, and non-parametric alternatives and transformations considered. Similar analyses will be performed stratifying by admission diagnosis (myocardial infarction, congestive heart failure, pneumonia). Tests of trend in proportions and longitudinal techniques will be used to compare measures taken over time (6- and 12-month follow-up). The two-sided test of proportions will be employed to compare the biochemically-verified rates of abstinence from smoking at 6-month follow-up of the two treatment arms. Other analyses regarding biochemical validation will be performed as per common Consortium of Hospitals Advancing Research on Tobacco (CHART) protocol [19].

To investigate patients’ use of text messaging via mobile phones and interest in a cessation intervention using this technology, we will examine prevalence of regular text-messaging use and interest in counseling delivered via text messaging. Descriptive analysis techniques and tests of proportions will be used to present and compare baseline characteristics of participants in the sub-study within each center.

## Discussion

In this study, we plan to compare the effectiveness and cost-effectiveness of two interventions to promote smoking cessation among hospitalized patients at urban public hospitals, focusing on the post-discharge period. By design, these interventions are easily generalizable and potentially implementable at a wide variety of health-care institutions. Our study targets a high-risk patient population, including patients with mental illness who have a particularly high prevalence of tobacco use.

In designing our study, we aim to balance the goals of broad inclusivity and generalizability with optimizing retention and follow-up rates in a difficult-to-reach population. Given that our intervention (counseling + medication) may be of benefit even in patients who seem unmotivated to quit smoking in the near future, we aim to include all smokers – irrespective of stated desire to quit. Similarly, since smoking cessation is likely to be beneficial to the health of all smokers and systems-level change is often implemented on a hospital-wide basis, we do not focus on patients in any one disease category – rather, the entire institutions are included.

The downside of aiming to be this inclusive in our entry criteria is that we will likely enroll participants who may be difficult to contact for follow-up post-discharge. The population of these hospitals (especially Bellevue’s) is highly mobile (both within New York City and around the world) and has proven difficult to maintain contact with in prior projects targeting patients post-discharge. Further, the smokers we enroll may be ambivalent about quitting smoking and also about participating in the study – making retention even more difficult. To enhance retention rates we have added brief follow-up calls at 2, 4, and 9 months post-discharge (in addition to the planned outcome measurements at 6 and 12 months post-discharge). During these brief interim calls, we will assess smoking status and confirm contact information (by contacting participants’ friends/family if needed).

Additionally, information garnered from this study will shed light on implementation concerns for programs of post-discharge contact with hospitalized patients. We will directly explore patients’ wishes regarding text messaging in comparison to telephone counseling. We will improve understanding of hospitalization as a true motivator for behavior change in the post-discharge period. Furthermore, we will uncover real-world concerns with accuracy of contact information, difficulties in reaching patients via telephone due to schedule, issues in applying standardized Quitline protocols to post-hospitalization patients, and adding a post-discharge component to usual hospital care. Since Joint Commission quality indicators for smoking cessation include post-discharge care [24] (and it is possible that they will extend this concept into other areas), systematic information about feasibility and implementation needs for such programs will be of use even outside the clinical area of smoking cessation.

Our study is subject to several limitations. Our primary outcome is based on self-report, which likely will falsely increase quit rates (as compared to biochemical verification). Given the mobility of our patient population and the fact that both of these hospitals are tertiary referral centers within their respective networks (thus potentially limiting post-discharge ongoing contact with patients), we will likely have significant dropout and inability to make contact to perform follow-up assessments. There is also some variability in ‘usual care’ experienced by the patients. However, we would expect all of these issues to be the same in both arms of the study, and the overall effect would be to decrease our likelihood of finding a difference between the arms. Also, we are limited by the services offered by our state’s Quitline: performing this study in a state with a Quitline similar to our in-house system might be less likely to find a significant difference between the two arms.

### Trial status

As of the time of manuscript submission, our trial is actively enrolling participants; we started enrollment in July 2011.

## Abbreviations

ANOVA: Analysis of variance; CHART: Consortium of Hospitals Advancing Research on Tobacco; EMR: Electronic medical record; NRT: Nicotine replacement therapy; QALY: Quality-adjusted life year; SP: Standardized patient; VA: Veterans Affairs.

## Competing interests

The authors declare that they have no competing interests.

## Authors’ contributions

All authors contributed to the design of the study and the preparation of the manuscript. SS, DS, and EG planned the study design and obtained funding. AG and EG are leading the team in implementing the study. IL planned and will perform the analyses related to effectiveness. RSB designed and will perform the cost-effectiveness analysis. ER designed and is implementing the telephone counseling program. All authors read and approved the final manuscript.
